# Heat transfer across a nanoscale pressurized air gap and its application in magnetic recording

**DOI:** 10.1038/s41598-018-21673-7

**Published:** 2018-02-20

**Authors:** Jinglin Zheng, Yung-Kan Chen, Qin Zhou

**Affiliations:** 1 Department of Mechanical and Materials Engineering, University of Nebraska at Lincoln, Lincoln, Nebraska 68588 USA; 2 Western Digital Corporation, San Jose, California, 95138 USA

## Abstract

In this study, we investigated how a thermally actuated air bearing slider heats up a fast-spinning storage disk through a highly pressurized nanoscale air gap in a magnetic recording system. A Eulerian-description-based computational approach is developed considering heat conduction through a pressurized air film and near-field radiation across the gap. A set of field equations that govern the air bearing dynamics, slider thermo-mechanics and disk heat dissipation are solved simultaneously through an iterative approach. A temperature field on the same order as the hot slider surface itself is found to be established in the disk. The effective local heat transfer coefficient is found to vary substantially with disk materials and linear speeds. This approach quantifies the magnitude of different thermal transport schemes and the accuracy is verified by an excellent agreement with our experiment, which measures the local slider temperature rise with a resistance temperature sensor. It also demonstrates an effective computational approach to treat transient thermal processes in a system of components with fast relative speed and different length scales. Finally, the investigated thermal transport mechanism leads to a substantial spacing change that has a significant impact on the spacing margin of today’s magnetic storage systems.

## Introduction

Heat transfer across a nanoscale gap is of fundamental importance in thermal management of micro and nanoscale devices. For example, in a magnetic recording system a slider built with magnetic read/write transducers “flies” above a fast-rotating storage disk by forming a highly pressurized air gap (air bearing). A tight magnetic spacing (i.e., the distance between the magnetic transducers and the recording layer of the storage disk) is necessary for higher data storage capacity^1^. For that purpose, thermal flying-height control (TFC) has been invented^2^, where a joule heater is embedded close to the magnetic transducers in the slider body. The heater induces thermal expansion so that the slider’s bottom surface (the so-called air bearing surface, or ABS) protrudes toward the disk and reduces the local magnetic spacing. The pressure in the air gap can build up to more than 100 atm, resulting in a strong thermal conduction flow directed from the slider to the disk. This process has received extensive investigation due to its strong coupling to the slider thermo-mechanics and the air bearing dynamics, resulting in significant impacts on crucial performances such as recording spacing, data storage density and power consumption^3–8^. With the rapid growth of storage capacity, the minimum gap size now approaches ~1 nm or even sub-nanometer. This nanoscale gap provides an opportunity to study thermal transport schemes between two surfaces separated by very small (<20 nm) distances, which is difficult to achieve owing to numerous technical challenges in creating and stably maintaining such gaps while simultaneously measuring small heat fluxes across the gap^9–11^. In the meantime, we also noticed a growing discrepancy between measured slider temperature fields and the predictions from conventional models used in the magnetic recording industry as the gap size decreases. Here we investigate the heat transfer across the nanoscale gap and find that (1) the small gap size and large heat flow changed the widely adopted ideal heat sink assumption on the fast spinning disk, which in return affected the slider’s mechanics and dynamics; (2) near field radiation, which happens between two surfaces with different material configurations, remains insignificant compared to thermal conduction through the pressurized gas film, even when the minimum gap size is only ~1 nm. Here we present an improved model which solves the disk temperature field established by the heat flow across the pressurized gap by formulating the transient process into a diffusion-advection-type equation using the control volume approach. This solution is then coupled with the slider thermo-mechanics and air bearing dynamics through thermal boundary conditions to solve the whole problem. The new model shows a substantial improvement over the conventional models in slider temperature predictions and exhibits an excellent agreement with experimental data.

The disk is in general considered as an ideal heat sink^12–14^ due to its much larger size compared to the slider and its high spinning speed (linear speed 10–30 m/s), quickly bringing fresh surfaces below the slider. This assumption is reasonable when we have a substantially-sized (>10 nm) air gap but encounters critical challenges when the minimum gap size is reduced to sub-nanometer^15,16^. In fact, there are experimental proofs that disk materials have a notable effect on the slider’s temperature field locally measured by an embedded contact sensor (ECS)^17,18^, which challenges the ideal heat sink assumption. However, so far there is no modeling approach that quantifies this disk temperature field and its subsequent impact on the recording system. One particular difficulty is that the spinning disk heats up as it moves underneath the slider and cools off as it moves out of the slider-covered area, making this a transient process that is difficult to fit into the widely-adopted steady-state approach. In this study we developed a Eulerian-description-based approach to characterize the disk heat dissipation with contributions from multiple thermal transport schemes at nanoscale, including the air film conduction and near field radiation. The model is then integrated into the multi-physics system and solved simultaneous with other field equations that govern the air bearing dynamics and slider thermo-mechanics with an iterative approach. The accuracy of the numerical model is confirmed by the excellent agreement with the experimental data. The disk temperature field established by the thermally-actuated slider is quantified to be at the same order as the heat source surface itself. Disk configuration and relative linear speed contribute significantly to the effective heat transfer coefficient across this interface and resultant temperature fields on both sides of the interface. This approach verifies the magnitude of different thermal transport schemes across a pressurized narrow gap maintained by air bearing dynamics. It also demonstrates an effective computational approach to treat transient thermal process in a system of components with fast relative speed and different length scales. It is also shown that for a magnetic recording system, the investigated thermal transport mechanism leads to a substantial spacing change of several angstroms, which account for a large part of today’s operating spacing (usually around 1 nm).

### Numerical model

**Figure 1:**
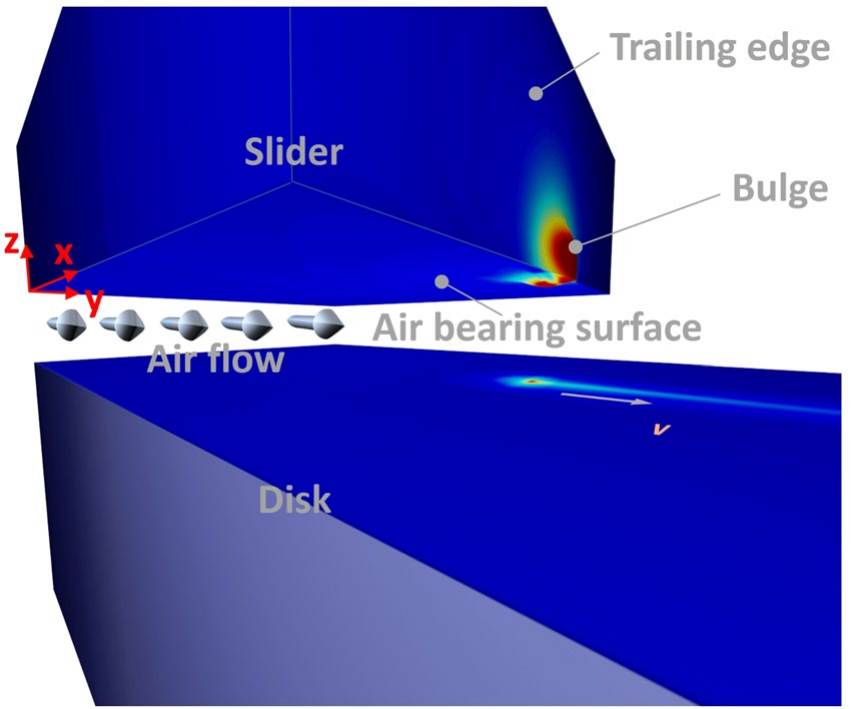
Nanoscale heat transfer at a slider-disk interface: air flow is brought into the gap by the fast spinning disk and get compressed by the air bearing surface. This forms a pressure field which keeps the slider at desired attitudes for read/write operations. A bulge is formed at the trailing edge of the slider by Joule heating to bring the read/write transducers closer to the disk. The hot slider surface can heat up the disk surface through this pressurized air gap. The disk temperature field is coupled to the slider’s thermo-mechanical deformation and the pressure field of the gap air flow, hence has to be solved simultaneously with other field equations.

The problem is formulated as shown in Fig. 1. The spinning disk brings air flow underneath the patterned ABS at the slider’s bottom, forming a pressure field which keeps the slider at desired attitudes, usually quantified by the slider’s flying-height, pitch and roll. A resistor heater built in the slider body locally heats up the slider and forms a bulge at the slider’s trailing edge to bring the recording transducers even closer to the disk. The minimum gap size between the slider and disk can reach 1 nm or even sub-nanometer. As seen, the geometry of the bulge determined by the thermo-mechanical deformation of the slider affects and is affected by the underlying pressure field. Now if the disk heats up and its temperature field becomes non-negligible, the heat flow between the slider and disk is expected to exert influences on the pressure field, the slider’s attitudes and the temperature/stress/strain field in the slider body.

Although the thermal process inside the disk is just a heat conduction problem governed by the heat equation, to integrate the disk temperature field into this coupled field analysis is not trivial. For one individual material point on the disk, it heats up as it spins underneath the slider and cools down as it moves out of the slider-covered area. The temperature varies periodically per revolution, and this transient process does not reach equilibrium. Considering the slider’s dimension, which is less than a hundredth of the disk’s circumference, the computational effort to fully simulate this temperature field is expected to be tremendous because it is critical to characterize the temperature field on the slider’s surface precisely. However, the disk, though large in its dimension, has uniform material configurations and properties throughout the *x*-*y* plane^19^. Therefore, if we change our perspective from the disk to the slider, namely, if we fix the coordinates with the slider, this disk temperature field does reach a steady state (i.e. temperature field in this coordinate does not change with time), as illustrated in Fig. 1. Therefore, we need to re-formulate the heat equation for the disk temperature field using Eulerian coordinates.

Using a control volume formulation combined with Fourier’s law, we first re-formulate the heat equation that governs the disk temperature field into a steady-state advection-diffusion-type equation:

1 $$\frac{\partial }{\partial x}(C{T}_{d}u-K\frac{\partial {T}_{d}}{\partial x})+\frac{\partial }{\partial y}(C{T}_{d}v-K\frac{\partial {T}_{d}}{\partial y})+\frac{\partial }{\partial z}(-K\frac{\partial {T}_{d}}{\partial z})=0$$

Here, ${T}_{d}(x,y,z)$ is the disk temperature represented in Eulerian coordinates, *C* and *K* are the volumetric heat capacity and thermal conductivity of disk materials, respectively, *u* and *v* are the down-track and cross-track linear speeds respectively. Disk vibrations in the *z* direction is neglected here. To solve equation (1), we developed a finite-volume-based numerical code with adaptive mesh in both lateral(*x* and *y*) and vertical (*z*) directions. In the lateral direction, the mesh in the area right beneath the slider matches with the mesh on the ABS to capture the lateral pressure gradient (which is closely related to the heat transfer coefficient) and avoid numerical errors introduced by interpolations. The temperature gradient in *z* is expected to drop steeply at the top solid layers because of no material movement. Therefore, the mesh size is adaptive to the temperature gradient and ranges from ~1 nm at the disk top to ~100 µm at the disk bottom in order for both accuracy and efficiency (see supplementary Fig. S1 details). Instead of solving the temperature of the entire disk, we confine our solution domain within a disk block sized at 1.7 mm × 0.7 mm × 0.8 mm starting from the leading edge of the slider and ending at the spatial location one-slider-length down-track from the slider’s trailing edge, to keep the problem at a moderate size. The boundaries of the solution domain are determined from a series of simulations with different domain sizes which confirms that a Dirichlet boundary condition at the ambient temperature *T*_0_ is appropriate for the side and bottom surfaces of the disk block.

*T*_*d*_ solved from equation (1) is coupled to the whole system through thermal flux boundary condition, which is determined from the slider’s temperature field as well as the air bearing dynamics. The pressure field between the slider and disk is solved from the generalized Reynolds equation, an adapted form of the classical Reynolds equation derived from a linearized Boltzmann equation, to accommodate the case of a highly rarefied air flow such as the pressurized thin film air bearing in our problem^20^. Given the load applied on the slider and the ABS geometry design, the pressure field as well as the slider attitudes are solved from the coupled Reynolds equation and the slider statics equation using CML Air^21^. These two solutions (spacing *d* and pressure *p*) are then related to the slider’s deformation under Joule heating through the heat flux boundary conditions at ABS:

2 $$q={q}_{cond}+{q}_{visc}+{q}_{rad}$$

As indicated by equation (2), three major thermal transport schemes exist, including conduction *q*_*cond*_, radiation *q*_*rad*_ and viscous heating *q*_*visc*_ from the air bearing. *q*_*cond*_, which is generally considered to be the dominant heat transport scheme and has been thoroughly studied^22–25^, can be obtained by solving the continuum energy equation with jump boundary conditions^22^, a very similar treatment just like the generalized Reynolds equation, and written in the following form:

3 $${q}_{cond}(x,y)=K\frac{{T}_{s}(x,y)-{T}_{d}(x,y)}{d(x,y)+2\frac{2(2-\alpha )\gamma }{\alpha (\gamma +1)Pr}\lambda (x,y)}$$

where *x* and *y* stand for the spatial coordinates along down-track and cross-track directions, respectively, as shown in Fig. 1. *K* is air thermal conductivity, *α* is the thermal accommodation coefficient, *γ* is the specific heat ratio, Pr is the Prandtl number, *T*_*s*_ is the slider’s local temperature at the ABS, *T*_*d*_ is the disk’s local temperature at the surface, which is solved from equation (1), *d* is the local spacing between the slider and the disk solved from CML Air. *λ* is the local air mean free path which is proportional to the film temperature *T*_*film*_ and inversely proportional to film pressure *p*(*x*, *y*). *p* is solved from the Reynolds equation and *T*_*film*_ is calculated as an average of *T*_*s*_ and *T*_*d*_, i.e., *T*_*film*_ = (*T*_*s*_ + *T*_*d*_)/2.

With the nanoscale gap size, the contribution from viscous heating becomes negligible^22^, whereas the radiative thermal conductance may become a notable contributor. To evaluate *q*_*rad*_, we adopt the theoretical model that extends the classical Planck’s law into the near field radiation regime^26,27^ and demonstrates qualitative agreement with experiments conducted using a real magnetic head^28^. In the studied temperature range, the model shows that the radiative heat transfer coefficient *h*_*rad*_ (defined by *q*_*rad*_/(*T*_*s*_ − *T*_*d*_)) is a strong function of the spacing d, in contrast to its weak dependence on *T*_*s*_ and *T*_*d*_^28^. Therefore, only the linear term is included in *q*_*rad*_ calculation to reduce the iteration costs.

A fixed-point iteration strategy is adopted in the main program where the slider’s thermal deformation is first solved in the commercial software ANSYS. The displacement solution is used to calculate the air bearing dynamics with CML Air and the temperature solution is used to simulate the disk temperature field with our finite volume solver. The obtained pressure and disk temperature fields are then applied in equation (2) to define a new set of thermal flux boundary conditions to be applied in the ANSYS model. This simulation strategy is then applied to examine the gap heating effect with two commonly-used disk types in the magnetic recording industry, namely, disks with aluminum and glass substrates. Material properties of both the slider and disk used in this analysis can be found from recent literature^13,29^.

## Results

### Established disk temperature fields

**Figure 2:**
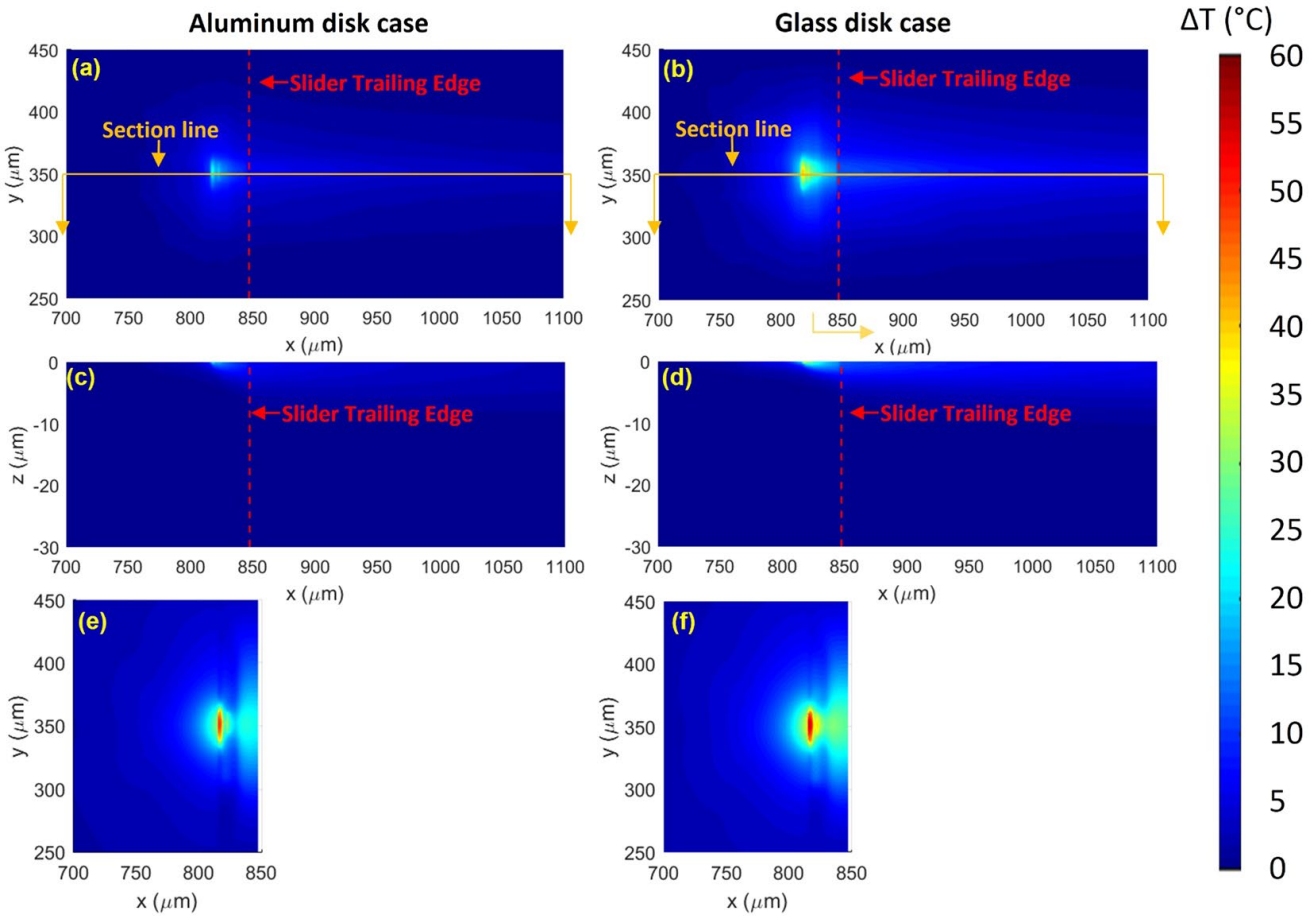
(**a**) Disk temperature rise in the aluminum case (*x-y* view). (**b**) Disk temperature rise in the glass case (*x-y* view). (**c**) Disk temperature rise in the aluminum case (*x-z* view). (**b**) Disk temperature rise in the glass case (*x-z* view). (**e**) Slider temperature rise in the aluminum case (*x-y* view). (**f**) Slider temperature rise in the glass case (*x-y* view).

Figure 2 shows simulated slider and disk temperature fields for the aluminum disk case (left) and the glass disk case (right) when 80 mW power is applied to the resistor heater inside the slider. Note that both disks have exactly the same slider temperature field and air pressure field as inputs in this case study. As shown in Fig. 2(a,b) a notable disk temperature field is established in both cases. The disk heats up as it moves underneath the slider and approaches the trailing edge, then cools off as it moves beyond the slider-covered area. The disk hot spot is stretched in the *x* (down-track) direction due to the spinning motion whereas its dimension in the *y* (cross-track) direction remain confined because heat dissipates only through thermal diffusion along *y*. As indicated by the color scale on the right, maximum disk temperature rise, Δ*T*_*d*_, is 24 °C for the aluminum disk whereas 42 °C for the glass disk, mostly due to glass’s small thermal conductivity. The *x*-*z* section views of the disk cut at the cross-track centerline (indicated in Fig. 2(a,b)) shows how the heat penetrates into the disk. Note that *z* = 0 is aligned with the disk top surface. The disk temperature decreases fast with depth and the heating effect is almost negligible beyond 10 μm into the disk.

Such a substantial difference in Δ*T*_*d*_ between the two disk types is expected to affect the temperature of the heat source, the slider, in return. Figure 2(e,f) show the temperature fields on the ABS for both cases, and the difference, though not as significant as Δ*T*_*d*_, is still substantial. Maximum Δ*T*_*s*_ in the glass disk case is 55 °C, 13% higher than 49 °C in the aluminum disk case. Considering that the current operating spacing in magnetic recording is ~1 nm, this temperature difference is expected to cause a notable change in the read/write spacing. It should also be noted that Δ*T*_*d*_ is at the same order as Δ*T*_*s*_, showing the heating mechanism across this pressurized gap is indeed very effective.

### Validity of the model

To check the validity of the model, it is crucial to measure Δ*T*_*d*_, which is very difficult to obtain at the operating conditions involving fast disk movement^30,31^. However, as shown in Fig. 2(e,f), Δ*T*_*d*_ does induce a quite notable change in Δ*T*_*s*_, and this can actually be obtained with an embedded contact sensor (ECS) built within the slider body. ECS is a temperature sensor originally designed to detect the contact between the slider and the disk^32^. However, the sensitivity of its resistance to the slider’s local temperature can be utilized to quantify Δ*T*_*s*_ at a specific location on the ABS, a piece of information that can be directly compared with our model prediction.

**Figure 3:**
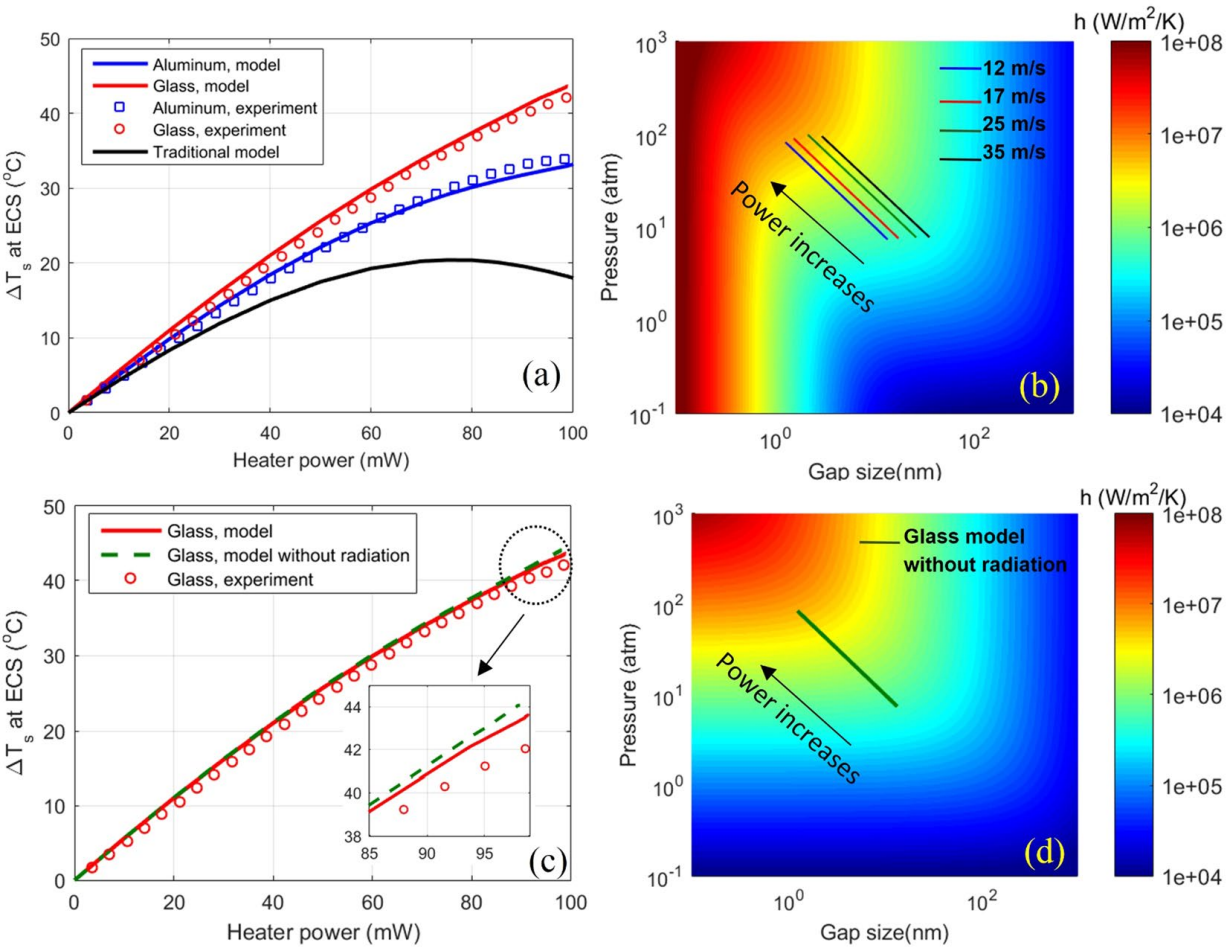
(**a**) Δ*T*_s_ at the ECS location with increasing heater power: experiment – model correlation. Prediction of the traditional model is also included for comparison. (**b)** Heat transfer coefficient *h* as a function of gap size *d* and pressure *p*. The four curves mark the relations between *p* and *d* under different linear speed for the studied ABS. The arrow marks the direction of increasing heating power where *d* decreases and *p* increases. (**c**) ΔT_s_ at the ECS location with increasing heater power – near field radiation effect. Near field radiation has minor impact compared to compressed gas conduction. (**d)** Heat transfer coefficient *h* as a function of gap size *d* and pressure *p* without considering near field radiation. Magnitude of *h* increases toward higher *p* and lower *d* and unlike (**b**), there is no radiation dominated area.

In Fig. 3(a), we show Δ*T*_*s*_ at the ECS location with increasing heater power and compare the model predictions and measurements side by side. A remarkably higher Δ*T*_*s*_ is observed in the glass case as compared to the aluminum case. The model predictions quantitatively agree with the measurements very well. To illustrate the significant impact of considering this disk temperature field, we also present Δ*T*_*s*_ predicted by the traditional TFC model taking the disk as an ideal heat sink, which is shown as the black curve in Fig. 3(a). This model is not only well off the measurements in terms of numbers, but also shows a trend of Δ*T*_*s*_ decreasing with power at the range of 80–100 mW, which obviously contradicts the experimental findings. Note that treating the disk as an ideal heat sink amplifies the disk-cooling effect on the slider. This effect grows with spacing reduction, and in the worst case, produces an artificial effect showing the disk-cooling overwhelms the heating on the slider, as indicated by the non-monotonic trend in the traditional model curve.

### Thermal transport across the pressurized nanoscale air gap

Compared to other nanoscale interfaces, the interface under study features a highly pressurized air bearing as a determinant factor in the thermal transport process. Therefore, it is interesting to quantify the magnitude of this interface heat transfer at different pressure levels and gap sizes. Figure 3(b) shows the heat transfer coefficient *h*, defined as the ratio between heat flux *q* and temperature difference (*T*_*s*_ − *T*_*d*_), as a function of the gap size *d* and the pressure *p*. At a big gap size, *h* is a strong function of both *p* and *d*, showing the heat transfer is dominated by the pressurized air film conduction. However, at ~1 nm gap size, *h* due to near field radiation reaches the order of 1 × 10^6^ W/m^2^/K, a magnitude comparable to *h* due to air film conduction. This change deforms the contour shape in Fig. 3(b): as *d* further reduces below 1 nm, the pressure becomes almost irrelevant and the gap size dominates, indicating the radiative flow overwhelms the conduction flow. The growth of *h* is fairly steep owing to the strong near field radiation effect^9–11^. For comparison, we also give a similar plot of *h* without considering near field radiation in Fig. 3(d), where we can observe a relatively slow transition of *h* with a very different pattern at low spacing (*d* < 1 nm). To further quantify how much the near field radiation scheme to the heat flow across the gap, we repeated the simulation of the glass case by taking out the near field radiation component in *h*. The result is plotted against the full model and experiment result in Fig. 3(c). The two model curves overlap very well, and gradually split toward high heater power (shown in the zoom-in figure) where the one with near field radiation is slightly closer to the measurement. The tiny scale of this split may look contradictive to the strong magnitude of near field radiation presented in Fig. 3(b). The reason is that the spacing profile is curved rather than flat (due to the bulge shape) and the near field radiation diminishes very fast with increasing spacing, meaning the area where strong radiative flow could occur is very small. Therefore, the integrated radiative heat flow has an overall negligible impact on the resultant slider temperature field.

It should be noted that once the ABS design is fixed, *p* and *d* are no longer independent variables but related through the Reynolds equation. Therefore, *h* degenerates into a curve on the surface map in Fig. 3(b), and this *h* curve is different at different locations. For example, *h* varies with *p* and *d* as marked by the blue solid line in Fig. 3(b) at the ECS location for the ABS being studied. As one can expect, a change in ABS geometry could modify the relation between *p* and *d*, hence the local heat transfer coefficient *h*, therefore, could be a possible method to fine-tune the thermal transport across this interface.

### Disk cooling effects

The heat transfer coefficient *h* doesn’t consider the effectiveness of the disk as a cooling medium. Here we further define an effective heat transfer coefficient *h*_*e*_ in equation (4). In the ideal case of the disk being a perfect heat sink, *h*_*e*_ is equal to *h*, which is a function of the air bearing pressure *p* and slider-disk spacing *d*. With the disk temperature increasing, *h*_*e*_ would deviate from *h*, and eventually diminish once the disk temperature rises up to the slider temperature, meaning the disk is no longer functioning as a cooling medium. Therefore compared to *h*, the value of *h*_*e*_ not only incorporates the impact from the air film interface, but also the effects arising from the disk side, including disk materials, configuration and linear speeds.

4 $${h}_{e}=\frac{{T}_{s}-{T}_{d}}{{T}_{s}-{T}_{0}}h$$

**Figure 4:**
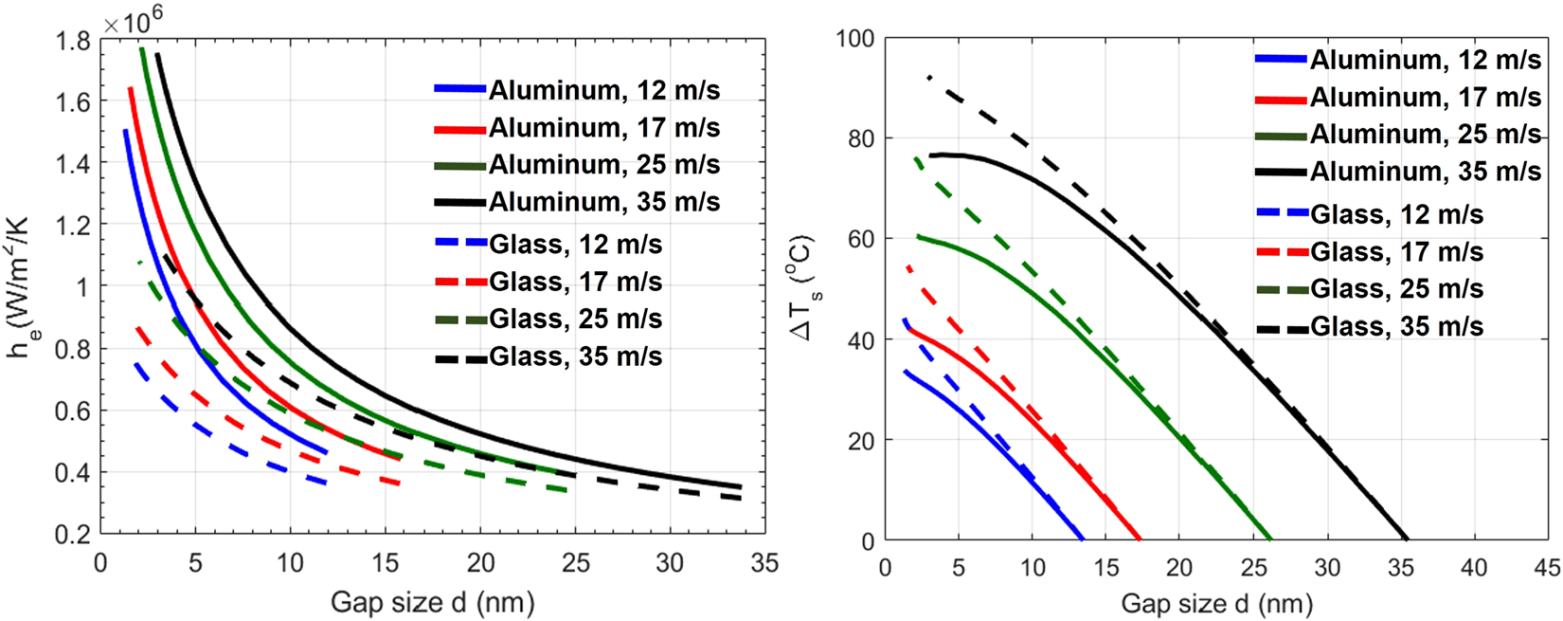
(**a**) *h*_*e*_ as a function of *d* for different linear speeds and disk materials. (**b)** Δ*T*_*s*_ as a function of *d* for different linear speeds and disk materials.

One unique feature of this pressurized gap, compared to other static systems, is that the nanoscale thermal transport can be modified by the disk linear speed *v*. Increasing *v* has a two-fold effect on the thermal transport. On one hand, it stiffens the ABS, leading to increased *p*, and accordingly, higher *h* at the same gap size *d*, due to a more-tightly packed air film. Figure 3(b) illustrates how the *h - p* - *d* curve shifts when *v* increases from 12 m/s to 35 m/s. The second effect emerges when the disk dissipates heat toward the environment. This process is governed by equation (1), where faster *v* shortens the heating cycle of the disk underneath the slider. Both effects are reflected in Δ*T*_*d*_, hence can be quantified by *h*_*e*_. In Fig. 4 (a), we compare *h*_*e*_ as a function of *d* for different linear speeds. Solid lines represent results using aluminum disks and dashed lines represent glass disks. In both cases, *h*_*e*_ increases significantly with *v* and the trend is consistent at all gap sizes and for both aluminum and glass disks.

However, this significantly increased *h*_*e*_ doesn’t really cool down the slider, which is shown in Fig. 4(b), where the Δ*T*_*s*_ actually increases with *v* if compared at the same *d*. This result seems contradictory to the analysis in Fig. 3(a) at first glance. The missing link is the provided power to heat the slider up and accomplish a certain gap size. As stated earlier, *d* reduction is realized by using a resistor heater to form a localized deformation. One side effect of this local deformation is that the air bearing pressure would build-up locally which pushes the slider away. This push-back effect from ABS dynamics grows with *v*, meaning that to reduce *d* to a certain number, more power has to be fed to the slider to compensate the push-back. Therefore, the trend in Fig. 4(b) is more of a result from the air bearing dynamics, which overwhelms the *h*_*e*_ effect in Fig. 4(a).

## Discussion

**Figure 5:**
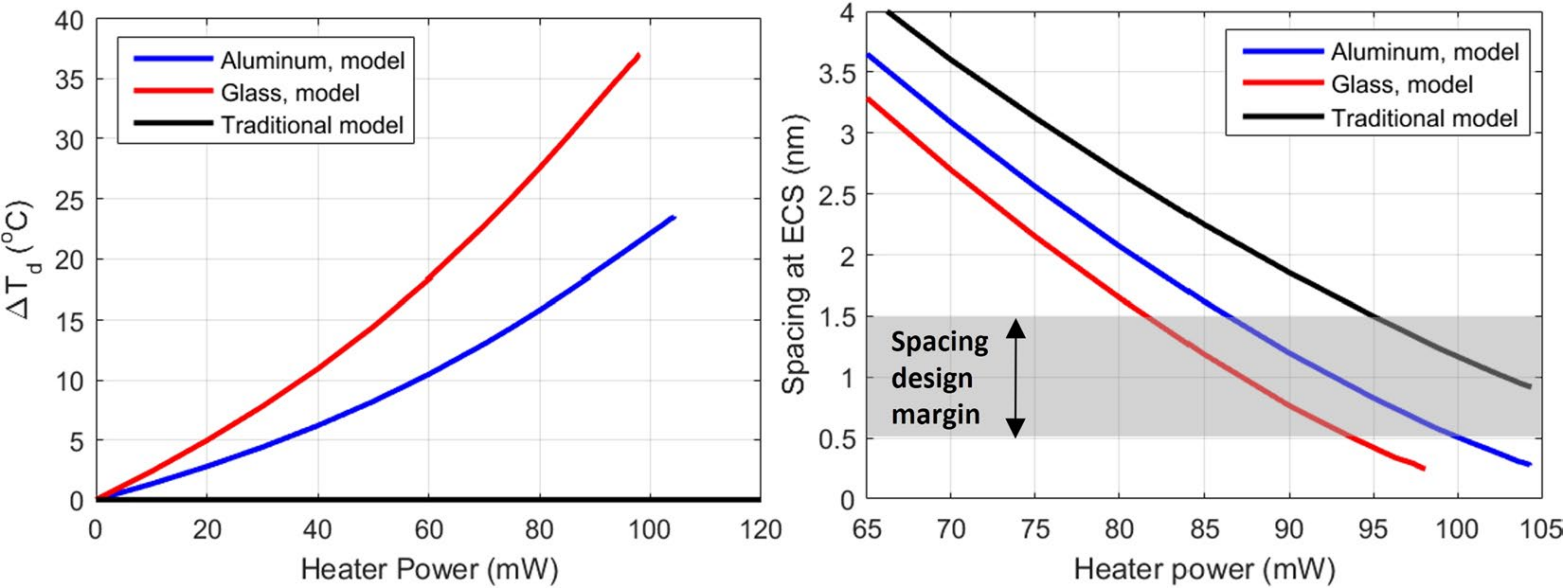
(**a**) Δ*T*_*d*_ at the disk location right underneath the ECS with increasing heater powers. (**b**) Spacing at the ECS location with increasing heat powers.

Finally, we will take a brief look at what this study implies on a magnetic recording system. Figure 5(a) compares the modeled Δ*T*_*d*_ at the disk location right underneath the ECS with increasing heater powers. Δ*T*_*d*_ increases monotonically with power and as expected, the glass case exhibits a much higher value. The magnitude of Δ*T*_*d*_, though significant enough, is not expected to cause big problems to the disk lubrication conditions or introduce severe disk local deformations, which are normally encountered in a heat-assisted-magnetic recording system^30^. On the other hand, the impact on the magnetic head performance is quite remarkable, in the sense that a remarkably lower spacing is established at the same power once Δ*T*_*d*_ is considered, as illustrated in Fig. 5(b). Today’s spacing design margin is mostly in the range of 0.5 nm–1.5 nm, as indicated by the shaded area in Fig. 5(b). Therefore, the spacing change due to Δ*T*_*d*_ actually accounts for a significant part of this very limited spacing margin (more than 50% if we ignore Δ*T*_*d*_ completely), hence has to be included into design considerations.

### Data availability

The datasets generated during and/or analyzed during the current study are available from the corresponding author on reasonable request.

## Electronic supplementary material

Supplementary information

## References

[1] Marchon B, Olson T (2009). Magnetic Spacing Trends: From LMR to PMR and Beyond. IEEE Trans. Magn..

[2] Shiramatsu T (2006). Drive Integration of Active Flying-Height Control Slider With Micro. Thermal Actuator..

[3] Shiramatsu T, Atsumi T, Kurita M, Shimizu Y, Tanaka H (2008). Dynamically Controlled Thermal Flying-Height Control Slider. IEEE Trans. Magn..

[4] Kurita M, Tokuyama M, Nakamoto K, Saegusa S, Maruyama Y (2005). Flying-height reduction of magnetic-head slider due to thermal protrusion. IEEE Trans. Magn..

[5] Juang JY (2008). Numerical and experimental analyses of nanometer-scale flying height control of magnetic head with heating element. IEEE Trans. Magn..

[6] Li H, Yin C-T, Talke FE (2009). Thermal insulator design for optimizing the efficiency of thermal flying height control sliders. J. Appl. Phys..

[7] Juang J-Y, Bogy DB (2007). Air-Bearing Effects on Actuated Thermal Pole-Tip Protrusion for Hard Disk Drives. J. Tribol..

[8] Liu N (2009). Thermal flying-height control sliders in hard disk drives filled with air-helium gas mixtures Thermal flying-height control sliders in hard disk drives filled with air-helium gas mixtures. Appl. Phys. Lett..

[9] Song B (2016). Radiative heat conductances between dielectric and metallic parallel plates with nanoscale gaps. Nat. Nanotechnol..

[10] St-Gelais R, Zhu L, Fan S, Lipson M (2016). Near-field radiative heat transfer between parallel structures in the deep subwavelength regime. Nat. Nanotechnol..

[11] Kim K (2015). Radiative heat transfer in the extreme near field. Nature.

[12] Juang J-Y, Bogy DB (2006). Alternate air bearing slider designs for areal density of 1 Tb/in/sup 2/. IEEE Trans. Magn..

[13] Zheng J, Bogy DB, Zhang S, Yan W (2010). Effects of altitude on thermal flying-height control actuation. Tribol. Lett..

[14] Liu, N., Zheng, J. & Bogy, D. B. Predicting the flying performance of thermal flying-height control sliders in hard disk drives. *J*. *Appl*. *Phys*. **108**, (2010).

[15] Li N, Zheng L, Bogy DB, Meng Y (2010). Flyability and Durability Test of Dynamic Fly-Height Sliders at 1-nm Clearance. Tribol. Trans..

[16] Xu, J., *et al* Contact/Clearance Sensor for HDI Subnanometer Regime. *IEEE Trans*. *Magn*. **50**, (2014).

[17] Peng, P., Johnson, M. & Stoebe, T. Storage Disk Substrate Effect On Dc And Ac Response Of Embedded Contact Storage Disk Substrate Effect On Dc And Ac Response Of Embedded Contact Sensor. In *Micromechatronics Inf*. *Precis*. *Equip*. (2015).

[18] Wu H, Xiong S, Canchi S, Schreck E, Bogy D (2016). Nanoscale heat transfer in the head-disk interface for heat assisted magnetic recording. Appl. Phys. Lett..

[19] Piramanayagam, S. N. Perpendicular recording media for hard disk drives. *J*. *Appl*. *Phys*. **102** (2007).

[20] Fukui S, Kaneko R (1988). Analysis of ultra-thin gas film lubrication based on linearized Boltzmann equation: first report—derivation of a generalized lubrication equation including thermal creep flow. J. Tribol..

[21] Lu SLS (1995). Air Bearing Design, Optimization, Stability Analysis And Verification For Sub-Micro-Inch Flying. IEEE Trans. Magn..

[22] Zhang S, Bogy DB (1999). A heat transfer model for thermal fluctuations in a thin slider/disk air bearing. Int. J. Heat Mass Transf..

[23] Chen L, Bogy DB, Strom B (2000). Thermal Dependence of MR Signal on Slider Flying State. IEEE Trans. Magn..

[24] Zheng H, Li H, Talke FE (2009). Numerical Simulation of a Thermal Flying Height Control Slider With Dual Heater and Insulator Elements. IEEE Trans. Magn..

[25] Zheng H (2010). The Effect of Thermal Radiation on Thermal Flying Height Control Sliders. IEEE Trans. Magn..

[26] Budaev BV, Bogy DB (2011). Extension of Planck’s law to steady heat flux across nanoscale gaps. Appl. Phys. A.

[27] Budaev BV, Bogy DB, Budaev BV, Bogy DB (2014). Computation of radiative heat transport across a nanoscale vacuum gap Computation of radiative heat transport across a nanoscale vacuum gap. Appl. Phys. Lett..

[28] Ma Y, Ghafari A, Budaev BV, Bogy DB (2016). Controlled heat flux measurement across a closing nanoscale gap and its comparison to theory. Appl. Phys. Lett..

[29] Suk M, Dennig P, Gillis D (2000). Magnetic Erasures Due to Impact Induced Interfacial Heating and Magnetostriction. J. Tribol..

[30] Xiong S, Bogy DB (2014). Investigation of the Local Temperature Increase for Heat Assisted Magnetic Recording (HAMR). IEEE Trans. Magn..

[31] Xiong, S. *et al* Thermal Response Time of Media in Heat-Assisted Magnetic Recording. *IEEE Trans. Magn*. **53**, 1–6 (2017).

[32] Shimizu Y (2011). Nano-scale defect mapping on a magnetic disk surface using a contact sensor. in IEEE Trans. Magn..

