# Supplementary material for: Heat transfer across a nanoscale pressurized air gap and its application in magnetic recording
Source: Sci Rep. 2018 Feb 20;8:3343. doi: 10.1038/s41598-018-21673-7 (PMC5820267; doi:10.1038/s41598-018-21673-7)
Supplement: Supplementary file 1 — Supplementary information [file 41598_2018_21673_MOESM1_ESM.pdf]

Heat transfer across a nanoscale pressurized air gap and its application in magnetic recording

Jinglin Zheng,[1*] Yung-Kan Chen,[2] and Qin Zhou[1]

[1]*Department of Mechanical and Materials Engineering, University of Nebraska at Lincoln, Lincoln, Nebraska 68588, USA*

[2]*Western Digital Corporation, San Jose, California 95138, USA*

Corresponding author: **jinglin.zheng@unl.edu, zhengjinglin0420@gmail.com**

Supplementary information

A finite volume scheme based on Patankar's formulation is developed to discretize equation (1). An upwind scheme is chosen for simplicity and numerical stability. Finite volume meshes in the lateral direction are carefully matched with the ABS grids to accurately capture the incoming heat flux, which is dependent on pressure gradient in the air film. Grid size in *z* direction is adaptive to the temperature gradient in z. Fig. S1 shows one example of disk temperature field varying along z-direction on grids of different sizes. It is evident that the disk temperature gradient is concentrated at the first a few microns. The adaptive mesh solution converges as the minimum mesh size dz_{min} is reduced below 100 nm. Results presented in the paper are obtained with dz_{min} = 20 nm.

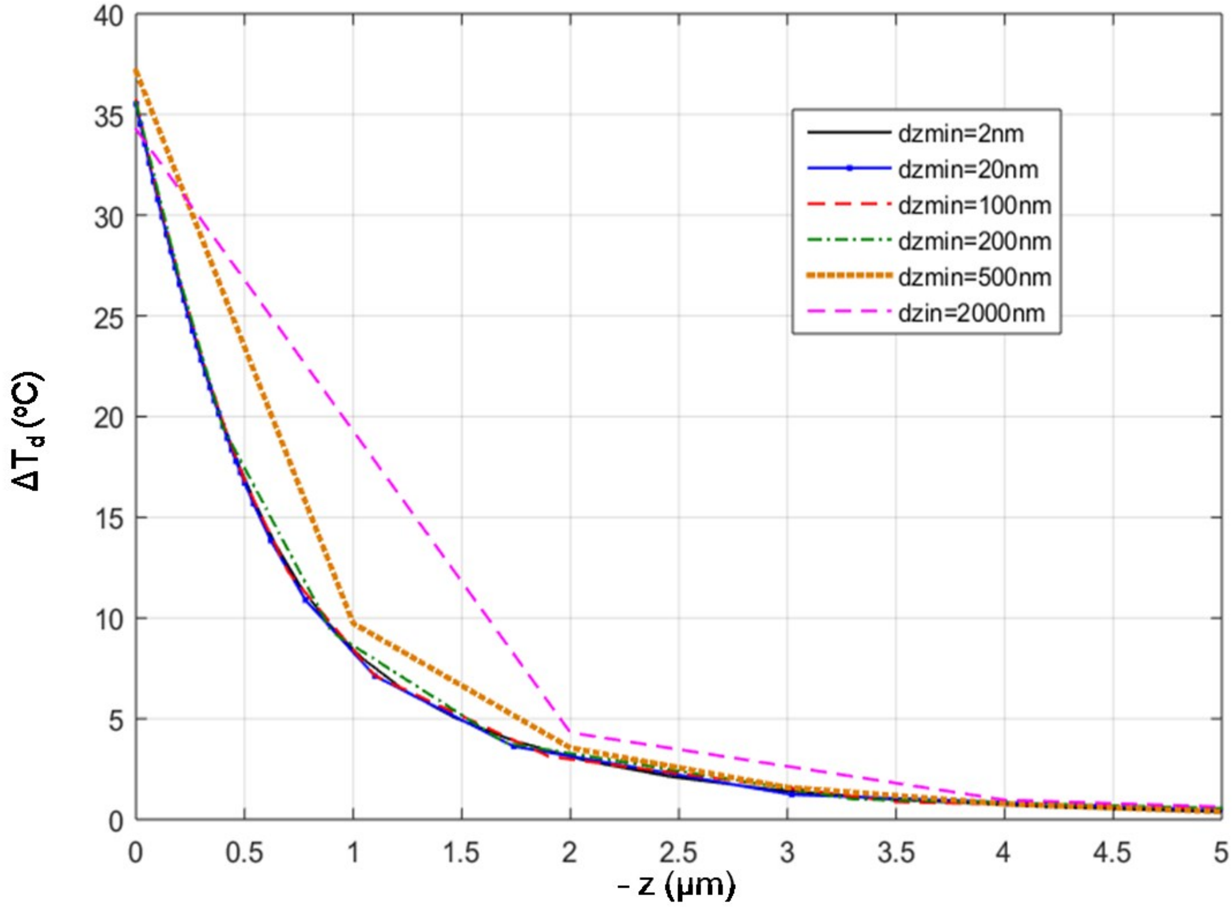

Figure S1 Solution convergence with z grid size reduction
